# Sustained poling-induced second-order optical nonlinearity in sodium-doped amorphous niobium oxide waveguides

**DOI:** 10.1038/s41598-026-45779-5

**Published:** 2026-03-26

**Authors:** Sirawit Boonsit, Lara Karam, Frederic Adamietz, Lydie Bourgeois, Milos Nedeljkovic, Nadege Courjal, Marc Dussauze, Ganapathy Senthil Murugan

**Affiliations:** 1 Optoelectronics Research Centre, University of Southampton, Southampton, SO17 1BJ UK; 2 Institut des Sciences Moléculaires, Université de Bordeaux, 33405 Talence Cedex, France; 3 FEMTO-ST TEMIS, 15B Avenue des Montboucons, 25030 Besancon Cedex, France

**Keywords:** Materials science, Optics and photonics, Physics

## Abstract

Nonlinear optical devices have attracted increasing interest, with lithium niobate being the benchmark material due to its strong second-order optical nonlinearity (SONL). However, its hardness, chemical inertness, and anisotropy present significant fabrication challenges, motivating the search for alternative platforms. Amorphous materials with poling-induced SONL offer promising opportunities, as their isotropic nature enables flexible optical design. In this work, we investigate amorphous sodium niobate thin films and demonstrate waveguide structures with active SONL through thermally controlled micrometer-scale poling combined with standard lithography. Using polarized second-harmonic generation (SHG) microscopy, we show precise spatial and geometrical control of the induced SONL, and its retention after waveguide processing steps, including UV lithography and dry-etching. The persistence of strong SHG signals, particularly near poling electrode edges, highlights the potential of aligning lithography mask with SONL patterns to enhance device performance, providing a foundation for developing efficient nonlinear photonic devices based on poled amorphous thin films.

**Supplementary Information:**

The online version contains supplementary material available at 10.1038/s41598-026-45779-5.

## Introduction

Materials with a second-order optical nonlinearity (SONL) find applications in various photonic devices, including high-speed modulators and frequency converters. This characteristic is typically observed in non-centrosymmetric materials, such as ferroelectric crystals like lithium niobate (LN), which exhibit a strong SONL response^1–3^. However, conventional bulk LN waveguides suffer from low refractive index contrast between core and cladding (≈ 0.02), leading to weak light confinement in the waveguide core. This limitation necessitates larger bend radii, making these waveguides impractical for complex devices or large-scale integration^4–7^. Over the past few years, LN-on-insulator (LNOI) has been developed to overcome this issue of low index contrast by performing ion slicing and bonding onto a low-index substrate (such as SiO_2_)^8–11^. However, the mechanical hardness, limited chemical reactivity, and anisotropic nature of LN impede precise etching at the micro- and nanoscale^12–14^. These challenges necessitate the exploration of alternative materials that offer more flexibility in fabrication^15–17^.

Amorphous materials with isotropic properties have emerged as promising alternatives, allowing for second-order susceptibility to be induced via thermal poling, as suggested by Myers et al.^18^. Later, Karam et al.^19,20^ published works on poled sodo-niobate thin films and demonstrated induced $\:{\chi\:}_{xxx}^{\left(2\right)}$ of 29 pm.V^−1^, which is the same order of magnitude as the SONL in bulk LN ($\:{{\chi}}_{\mathrm{zzz}}^{\left(\mathrm{2}\right)}\mathrm{=55}$ pm.V^−1^)^21,22^. With broad transparency and high refractive indices (2.1–2.2) from UV to mid-IR (0.35–5 μm)^19^, these materials are promising candidates for a nonlinear optical waveguide platform. Despite these advances, the fabrication of a niobate amorphous material with induced nonlinearity into waveguides and the stability of the induced nonlinearity after waveguide fabrication remain unexplored.

In this study, we investigate the robustness of the poling-induced nonlinearity in sodo-niobate thin films and determine the ideal placement and structuring of waveguides in these films to align with the peak induced SONL. The work covers five main aspects: (i) optimized deposition of sodo-niobate thin films via RF magnetron sputtering, (ii) thermal poling to induce accurate patterning of SONL, (iii) waveguide fabrication using UV optical lithography and an Ar-based plasma etching process, (iv) confirmation of the retention of the induced SONL after waveguide fabrication and (v) identification of the peak position of the induced nonlinear effect during structured electrode poling for precise placement of waveguide.

## Results and Discussion

### ***µ***-SHG characterization of poling-induced nonlinearity in sodo-niobate films

We first deposited the sodo-niobate (Na_2_O: Nb_2_O_5_) film onto a BF33 glass substrate using RF magnetron sputtering. The film was then thermally poled to break its centrosymmetric properties, leading to the induction of SONL. The poled sodo-niobate thin films were used to fabricate waveguides through standard UV contact lithography and Ar-ion beam milling, with additional fabrication details provided in the Methods section. Both the poled films and the subsequently fabricated waveguides were characterized using *µ*-SHG microscopy.

**Fig. 1:**
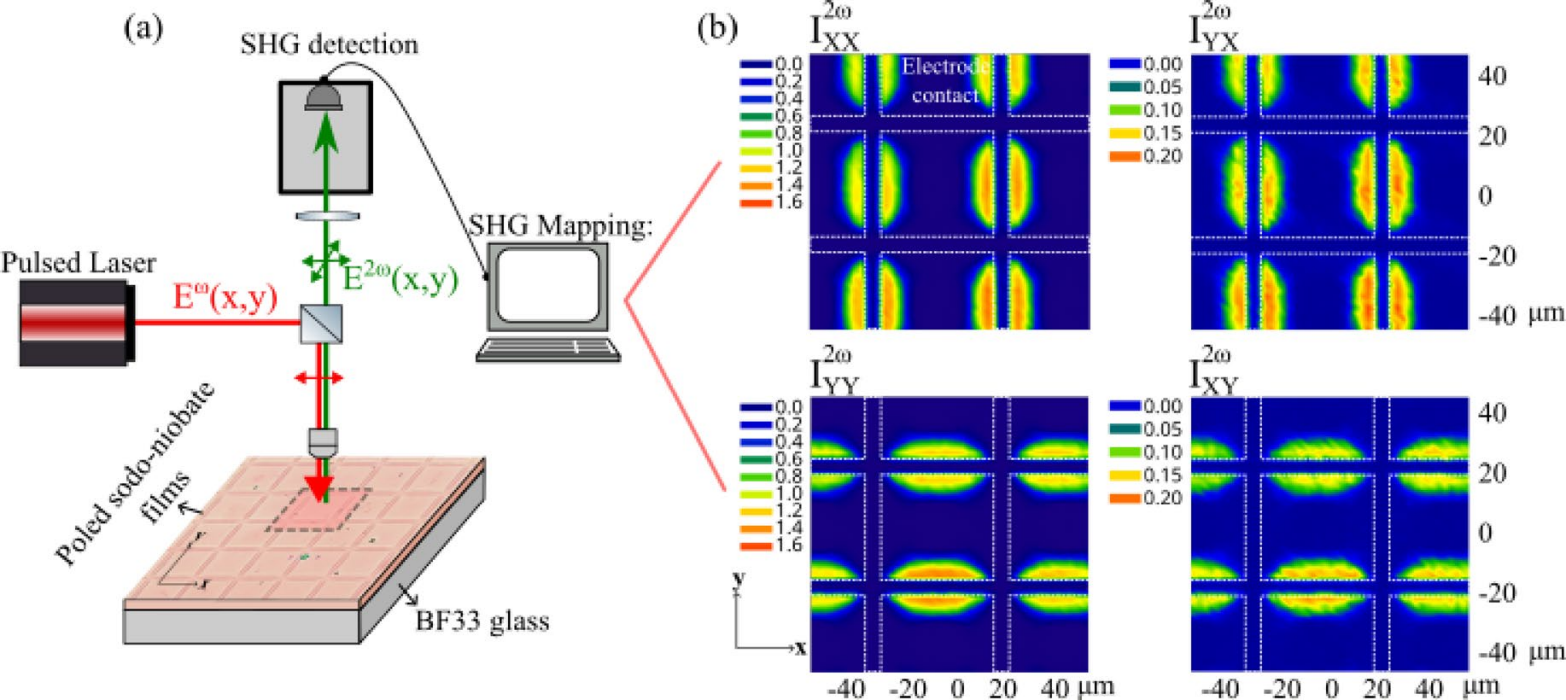
Schematic of ${\mu\:}$**-**SHG measurement on poled sodo-niobate films:**a** *µ*-SHG measurement apparatus **b** SHG maps of a 900 nm thick film containing 5 atomic% of Na and poled using 900 V and a 40 × 40 µm^2^ grid anode for different polarizations of incident light and SHG signal: $\:{I}_{XX}^{2\omega\:}$, $\:{I}_{YX}^{2\omega\:}$, $\:{I}_{YY}^{2\omega\:}$ and $\:{I}_{XY}^{2\omega\:}$.

A homemade polarized *µ*-SHG microscopy set up as shown in Fig. 1(a) was used to measure the spatial profiles of poled films by collecting polarized SHG in reflection mode. A detailed schematic is shown in Supplementary Fig. 1. A picosecond pulsed laser at 1064 nm was used with controlled linear polarization of the incident field. The laser beam was focused onto the film, and the resulting SHG signal reflected (epi-detection mode) from the film surface back towards the filters. Wavelengths other than the signal 532 nm component were filtered before reaching the photomultiplier tube. The polarized SHG signal measured is labelled as $\:{I}_{XX}^{2\omega\:},{I}_{YY}^{2\omega\:},{I}_{XY}^{2\omega\:}$ or $\:{I}_{YX}^{2\omega\:}$, where the first and second subscript refers to the incident laser and reflected SHG polarization states, respectively. By positioning the pattern of the poled films along the light polarization X and Y-axis, this methodology allows probing of the in-plane components ($\:x,y$) of the poling induced second-order nonlinearity $\:{\chi\:}^{\left(2\right)}$ tensor: $\:{I}_{XX}^{2\omega\:}{\propto\:\left({\chi\:}_{xxx}^{\left(2\right)}\right)}^{2}$, $\:{I}_{XY}^{2\omega\:}{\propto\:\left({\chi\:}_{yxx}^{\left(2\right)}\right)}^{2}$, $\:{I}_{YY}^{2\omega\:}{\propto\:\left({\chi\:}_{yyy}^{\left(2\right)}\right)}^{2}$ and $\:{I}_{YX}^{2\omega\:}{\propto\:\left({\chi\:}_{xyy}^{\left(2\right)}\right)}^{2}$.

**Fig. 2:**
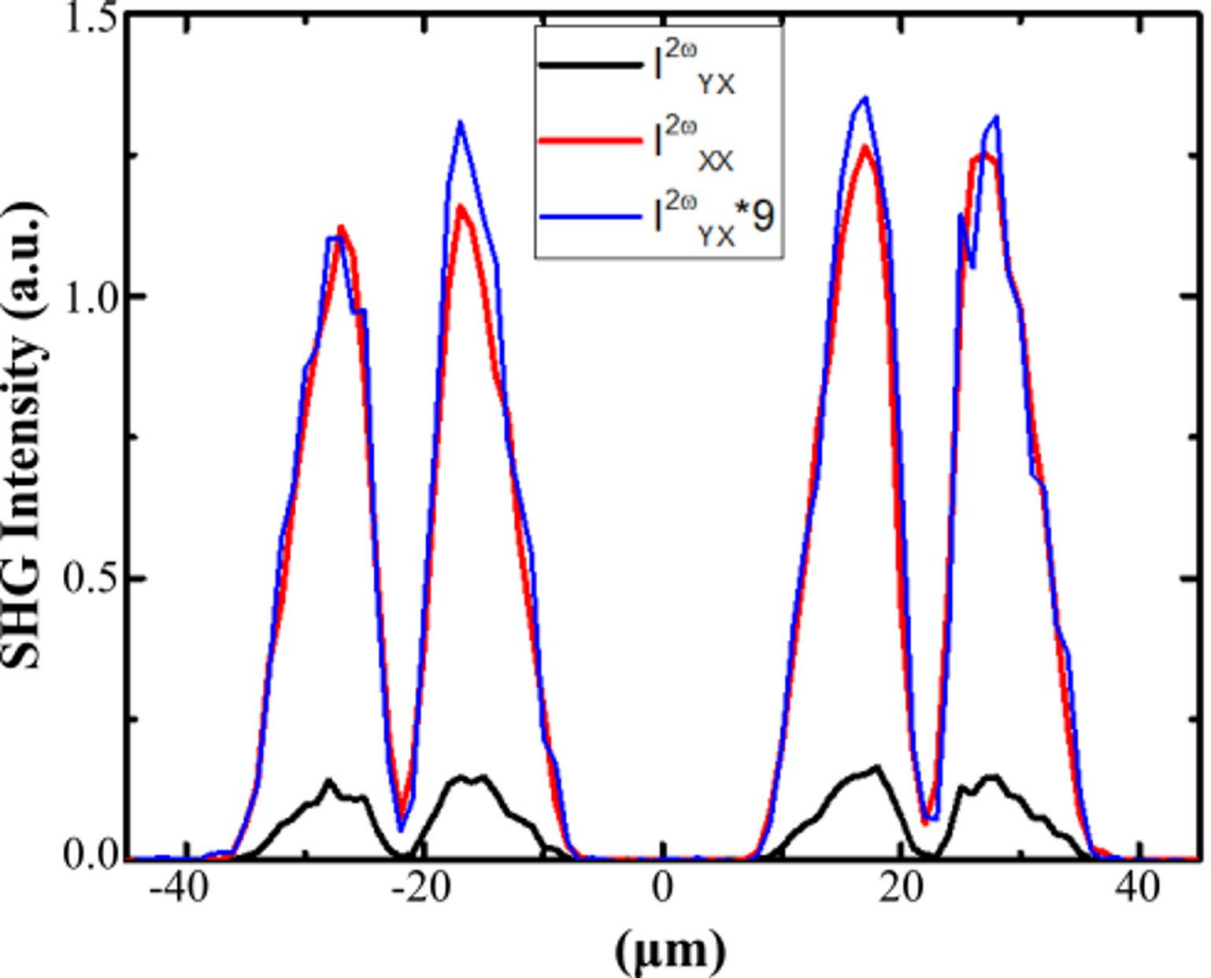
Comparison of ${\mu\:}$-SHG intensity for parallel and crossed polarizations. SHG profiles of $\:{I}_{XX}^{2\omega\:}$and $\:{I}_{YX}^{2\omega\:}$ extracted along the x-axis for y = 0 from the SHG images depicted in Fig. 1(b).

The SHG intensity maps presented in Fig. 1(b) were measured on a 900 nm thick sodo niobate film containing 5 atomic% of Na and thermally poled at 250 °C/900 V using a 40 × 40 µm^2^ grid electrode with 4 μm width. This measurement reveals spatially confined SHG signals localized near the edges of the grid electrode Pt strips. The patterned electrodes generate electric fields with both longitudinal and lateral components. The longitudinal field drives ionic migration through the film thickness, while the lateral field at the electrode edges enhances charge accumulation and distorts the local field. This leads to intensified electric fields and a strong in-plane SHG signal near the electrode edges^20,23^. Notably, for parallel polarizations, such as, $\:{I}_{XX}^{2\omega\:}$ or $\:{I}_{YY}^{2\omega\:}$, we can only detect the signal when the light polarization is perpendicular to the electrode edges. Similarly, for SHG measured in cross-polarizations, $\:{I}_{XY}^{2\omega\:},{I}_{YX}^{2\omega\:}$, the active zones are observed when the polarization of the SHG signal is perpendicular to electrode edges. One should notice that for the same SHG polarization state, a perfect co-localization of the signals measured in parallel and crossed polarization is observed allowing the polarization ratio $\:{I}_{XX}^{2\omega\:}/\:{I}_{YX}^{2\omega\:}$ and $\:{I}_{YY}^{2\omega\:}/\:{I}_{XY}^{2\omega\:}$ to be investigated, to get some insights about the relative values of the poling induced $\:{\chi\:}^{\left(2\right)}\:$tensor coefficients. In Fig. 2, SHG profiles extracted for *y* = 0 from Fig. 1(b) compare the relative intensities of $\:{I}_{XX}^{2\omega\:}\:\mathrm{a}\mathrm{n}\mathrm{d}\:{I}_{YX}^{2\omega\:}$ pointing out the ratio $\:{I}_{XX}^{2\omega\:}/\:{I}_{YX}^{2\omega\:}$ =9 (± 5%) (similar for $\:{I}_{YY}^{2\omega\:}/\:{I}_{XY}^{2\omega\:}$).

As anticipated by earlier research, these findings align with $\:{\chi\:}^{\left(2\right)}$mechanism caused by a poling-induced loss of centrosymmetry. In this process, the second-order optical response arises from the interaction between a static electric field $\:{E}_{stat}$ and the third-order susceptibility $\:{\chi\:}^{\left(3\right)}$ of an isotropic medium, expressed as $\:{\chi\:}^{\left(2\right)}=3.{\chi\:}^{\left(3\right)}{E}_{stat}$. If the micropoling procedure induces a static field oriented along the x-axis (or y-axis) and the niobate film keep its amorphous nature and centrosymmetry, it imposes the relationship $\:{\chi\:}_{xxx}^{\left(2\right)}=3.{\chi\:}_{yxx}^{\left(2\right)}$ (or $\:{\chi\:}_{yyy}^{\left(2\right)}=3.{\chi\:}_{xyy}^{\left(2\right)})$ which is in accordance with the SHG intensity polarization ratio observed in Fig. 1(b) and Fig. 2. Finally, the polarized second-harmonic generation (SHG) measurements revealed spatially patterned multicomponent electric fields, with precisely controlled in-plane orientation and positioning. These localized polarization effects are likely tied to the directional modulation of surface electrical currents during the thermo-electrical poling process, enabling tailored control over both charge gradients and frozen static field geometries.

**Fig. 3:**
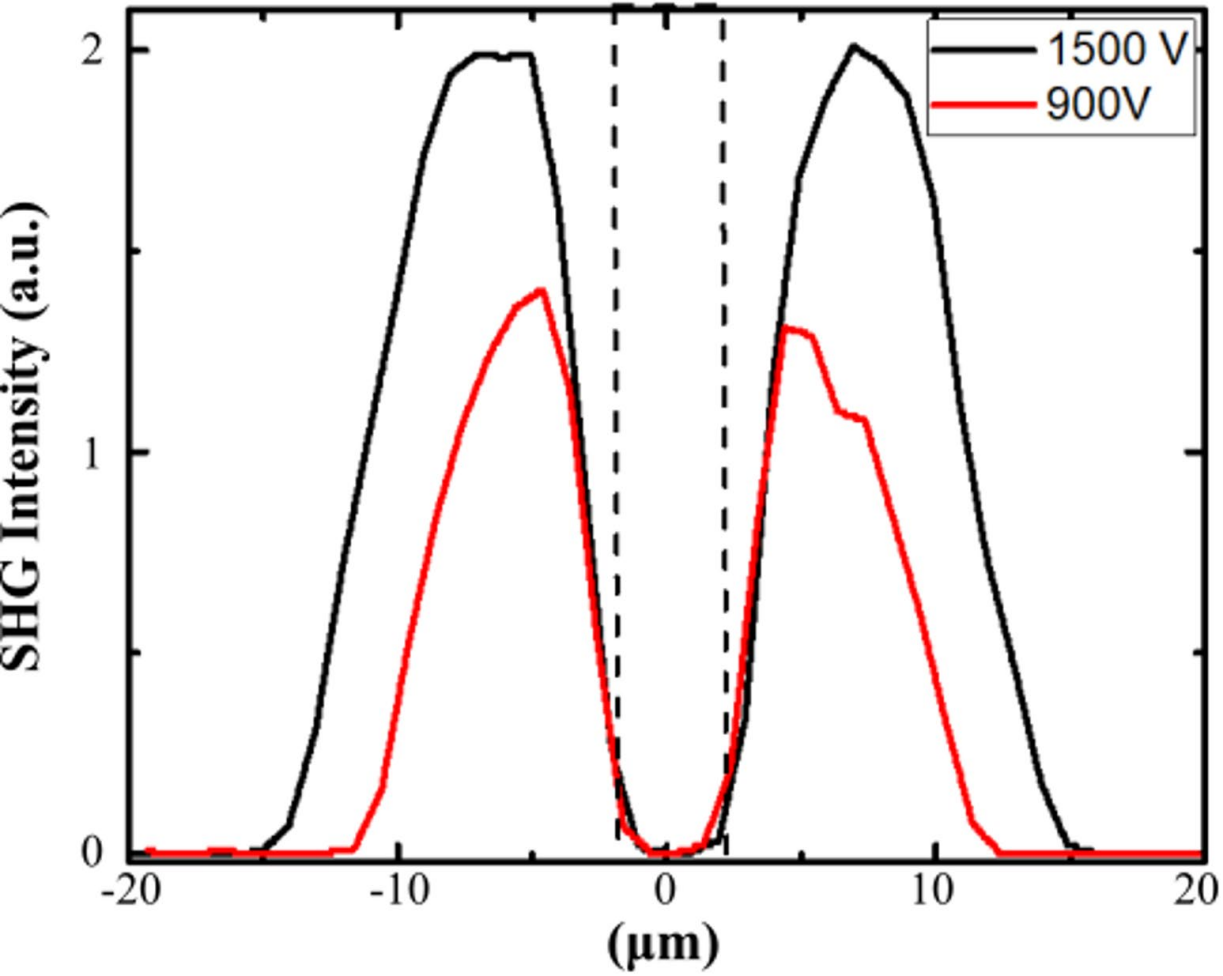
Influence of poling voltages on induced SHG signal. SHG intensity profiles across the electrode contact zone for two amorphous niobate films (500 nm thick containing 10 atomic% of Na) thermally poled using 1500 V and 900 V. The dashed line corresponds to the position of the anode 4-µm-wide Pt strip.

In the next step, we have evaluated the effect of the poling voltage on the poling induced SHG patterns. Two amorphous niobate thin films with the same sodium content (10 atomic% of Na) and the same thickness (500 nm) were thermally poled at the same temperature under two different applied voltages: 900 V and 1500 V. The electrodes for both samples consisted of a 70 × 70 µm^2^ grid. As shown in Fig. 3, we have analyzed the spatial profiles of SHG induced perpendicularly to the electrode edges, i.e. perpendicularly to the long axis of the 4-µm-wide Pt strip. The induced SHG signal was measured using parallel light polarizations, such as, $\:{I}_{XX}^{2\omega\:}$ with *X* aligned along the short axis of the electrode strip.

It confirms that the in-plane SHG signals are spatially confined close to the electrode edges. The shapes of the SHG profiles are similar for both voltages and are asymmetrical with a maximum of SHG intensity pointing at a distance of 4.5–7.0 μm from the center of electrode, and a full width at half maximum of 6 μm and 8 μm for poling voltages of 900 V and 1500 V, respectively. Increasing the poling voltage yields to stronger SHG intensities, with an amplitude increasing by a factor of 1.5 from 900 V to 1500 V. In our waveguide fabrication procedure, such preliminary characterizations are essential to accurately locate the optimal position for the etching process, as described in the next section. The SHG-active region is sufficiently broad to enable the fabrication of micrometer-scale waveguides suitable for single-mode optical guiding, as demonstrated in our previous study^24^. Moreover, these sets of data demonstrate that the micro-poling process allows (**i**) tuning the spatial SHG profile by adapting the poling voltage, and (**ii**) accurate control of the poling induced $\:{\chi\:}^{\left(2\right)}\:$tensor coefficients.

### ***µ***-SHG characterization of poling-induced nonlinearity of etched straight waveguides

In this section, we focus our attention on the fabrication of straight waveguides by etching procedures on thermally poled sodo-niobate thin films (more detail in the methods section). These waveguides were predominantly aligned in proximity to the electrode regions responsible for poling induced SHG responses. During the measurements, the orientation of the electrode edge was adjusted to be either perpendicular or parallel to etched structure. The $\:\mu\:$-SHG measurements were performed using the same procedure as described for poled thin films. The two parallel SHG polarization configurations will be employed, denoted as $\:{I}_{XX}^{2\omega\:}$, and $\:{I}_{YY}^{2\omega\:}$.

**Fig. 4:**
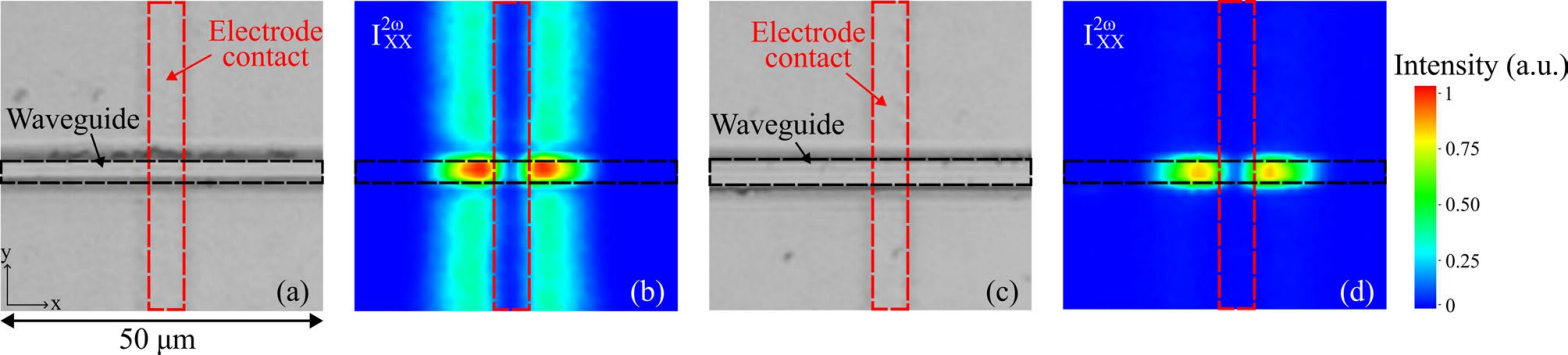
Precise spatial and geometric control of the SHG signal induced on a small etched waveguide. **a**, **c** Optical microscope images of the etched waveguides (indicated by black dashed lines), with the location of the poling electrodes contact (marked by red dashed lines). The collected signal of $\:{I}_{XX}^{2\omega\:}$ from **b** partially etched waveguides with 3 μm width and 500 nm thickness, **d** fully etched waveguides with 4.5 μm width and 900 nm thickness. Both films contain 5 atomic% Na and were poled under a poling voltage of 1500 V.

Figure 4 presents the results for both partially and fully etched waveguides, oriented perpendicular to the poling electrode. In this configuration, $\:{I}_{YY}^{2\omega\:}$​ signal was absent because the incident polarization aligned parallel to the electrode edges. Figure 4(b) and (d) compare the SHG intensity maps of $\:{I}_{XX}^{2\omega\:}$​ for the two waveguide structures. In both cases, the SHG response persists after etching and peaks at the waveguide position. Notably, a residual response remains along the electrode edge in regions where the film is only partially etched. By contrast, the fully etched sample shows $\:{I}_{XX}^{2\omega\:}$​ exclusively within the waveguide, with no detectable signal in the surrounding area where the film has been etched.

**Fig. 5:**
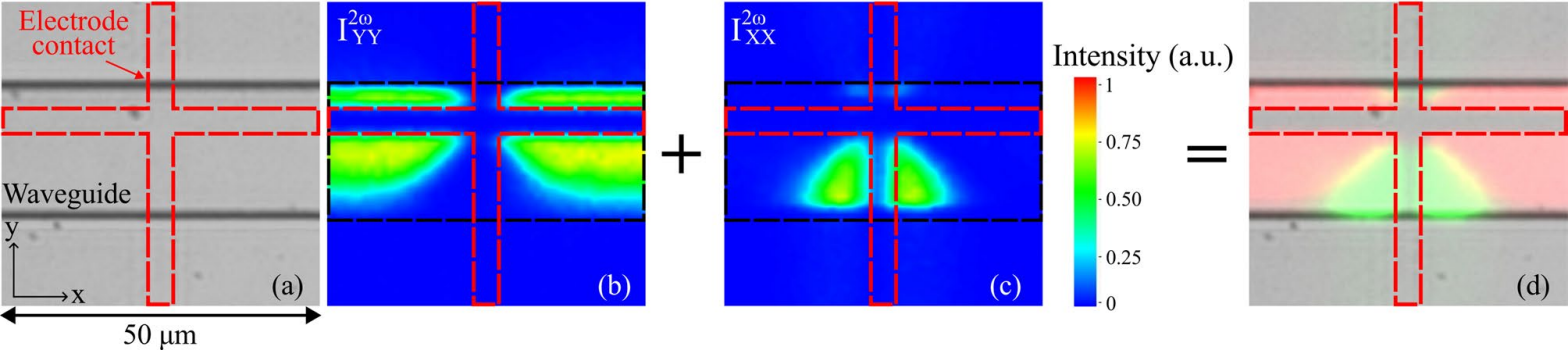
Precise spatial and geometric control of the SHG signal induced on a large etched waveguide. **a** Optical microscope image of a 20 μm wide and 500 nm thick waveguide poled under the poling voltage of 1500 V of 5 atomic% of Na film. The red dashed lines indicate the location of the poling electrodes contact. **b**,** c** Measured induced SHG signal: $\:{I}_{YY}^{2\omega\:}$, $\:{I}_{XX}^{2\omega\:}$, respectively. **d** The overlap of the $\:{I}_{YY}^{2\omega\:}$ (red) and $\:{I}_{XX}^{2\omega\:}$ (green) signals overlaid on the microscope image, indicating the spatial localization of the poling effect on the waveguide.

As a second example, to study the second order optical properties of a waveguide structure fabricated by etching process on thermally poled sodo-niobate thin films, a 20 μm wide straight channel was fabricated along the x-direction overlapping the poled region. It was chosen to be 20 μm wide in order to coincide with one intersection of the anode Pt grid used for the micro-poling treatment, as indicated by a red dashed line in Fig. 5(a). Both parallel components ($\:{I}_{YY}^{2\omega\:},{I}_{XX}^{2\omega\:}$) were observed in this fully etched linear structure, as shown in Fig. 5(b, c). The location of the horizontal and vertical SHG signals as a function of the position of the anodic grid intersection is in full agreement with the data of Fig. 1 (SHG patterning measured prior to the etching steps). Figure 5(d) presents the spatial overlap of the induced SHG signals originating from the vertically (in red, $\:{I}_{YY}^{2\omega\:}$) and horizontally (in green, $\:{I}_{XX}^{2\omega\:}$) poled zones. These results clearly illustrate that the precise geometrical control of the SHG response is preserved, as the etching process removes the surrounding regions of the film without degrading or altering the induced poling effects. In addition, the poled waveguides preserved their SHG response for over a year after fabrication, despite multiple processing steps – including UV lithography (resist baking), sputtering, dry etching – and exposure to temperatures up to 150 °C. These results indicate that the thermally poled structure is robust and retains its nonlinearity under typical fabrication and operating conditions.

In the final stage of this study, our objective was to optimize the second-order nonlinear optical response within the etched waveguide by determining the optimal lateral positioning of the waveguide relative to the electrode edge. To achieve this, we employed a 500 nm-thick amorphous niobate film containing 10 atomic% Na, thermally poled under an applied voltage of 1500 V. These conditions are identical to those used in the experiments presented in Fig. 3, which display the spatial distribution of the SHG signal after the poling process and prior to etching.

**Fig. 6:**
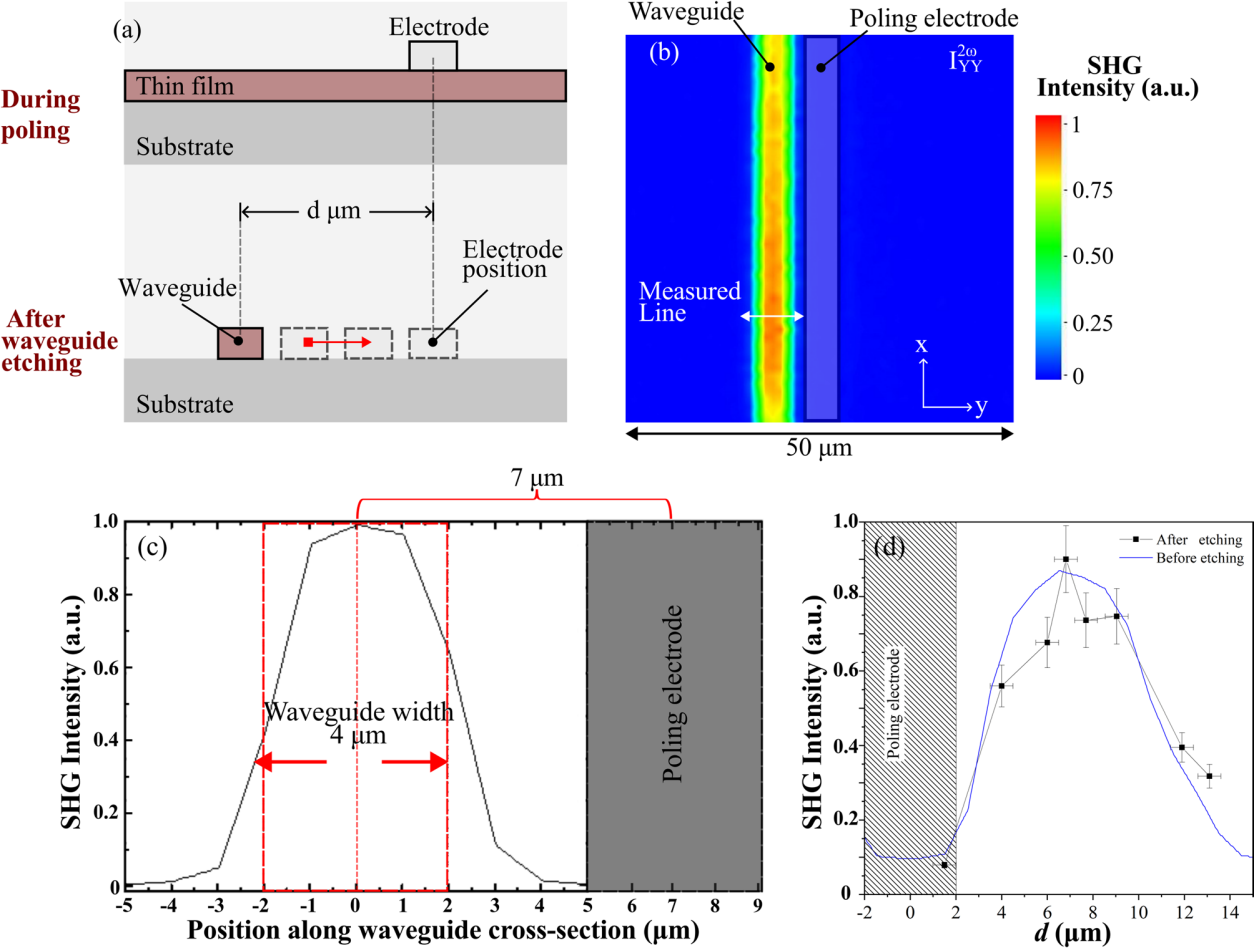
Optimizing poling electrode distance to etched waveguide. **a** Schematic of the protocol used to find the optimal position of the waveguide as a function of the distance from the center of electrode. **b**, **c** examples of SHG map and corresponding SHG profile obtained for the waveguide (center) positioned at a distance of 7 μm from the center of electrode. **d** Maximum SHG intensities measured in etched waveguides as a function of the separation between waveguide and poling electrode compared with the SHG profile measured before the etching procedures (extracted from Fig. 3).

As schematically illustrated in Fig. 6(a), the lateral distance (*d)* between the position of the waveguide center and the poling electrode center was systematically varied. For each configuration, a complete SHG mapping was performed in order to quantify the relative SHG intensity as a function of etching/waveguide position. Representative data are presented in Fig. 6(b) and Fig. 6(c), which respectively show an SHG intensity map and the corresponding $\:{\mathrm{I}}_{\mathrm{Y}\mathrm{Y}}^{2{\upomega\:}}$ intensity profile for a 4 μm-wide etched waveguide structure. The maximum relative SHG intensities obtained for each waveguide position (Supplementary Fig. 2) are plotted together in Fig. 6 (d) and compared with the SHG spatial profile recorded prior to the etching process (as shown in Fig. 3). The strong correlation between the pre- and post-etching SHG intensity profiles demonstrates that the poling-induced nonlinear optical properties of the amorphous niobate films are well preserved during the etching process. This result provides clear evidence of the compatibility of thermally poled amorphous niobate thin films with standard microfabrication techniques. Furthermore, these observations confirm that the optimal positioning of the waveguide can be accurately predicted from the SHG spatial profile measured before etching, i.e., following the micro-poling treatment. In the case presented in Fig. 6, the optimal distance was determined to be approximately 7 μm from the center of the electrode to the center of waveguide with 4 μm width. Taking into account the spatial resolution of the SHG microscope (approximately 1.5 μm), which introduces a convolution of the measured SHG profiles such as that shown in Fig. 6(c), it can be inferred that the fabricated waveguide structure exhibits a homogeneous second-order nonlinear optical response across its cross-section.

## Conclusion

This study demonstrates the robustness of the localized induced SONL effects in thermally poled sodo-niobate amorphous thin films on BF33 glass substrates after going through a waveguide fabrication process. Using RF magnetron sputtering, thin films with varying sodium concentrations were deposited, followed by thermal poling and waveguide fabrication via UV optical lithography and Ar-based plasma etching. The persistence of the induced nonlinearity was evaluated through polarization-controlled *µ*-SHG measurement, focusing on linear light polarization to probe the in-plane components of induced $\:{\chi\:}^{\left(2\right)}$. The results confirm that the induced SHG persists in the fabricated sodo-niobate waveguides, which allows the second-order nonlinear optical response in the fabricated waveguide structure to be optimized via precise alignment of the lithography mask to the poled pattern. These findings establish the feasibility of integrating poling-induced SONL in amorphous materials for practical nonlinear photonic applications, including electro-optic modulators and Mach-Zehnder interferometer (MZI)-based spectrometers. This work paves the way for future research and development in amorphous waveguide platforms for nonlinear optics.

## Methods

### RF magnetron sputtering deposition of sodo-niobate thin films on BF33 glass substrate

The sodo-niobate thin films (with thicknesses of 500 nm and 900 nm) were deposited onto clean borofloat33 (BF33) glass substrates using a radio frequency (RF) magnetron sputtering system (AJA International Orion Series Sputtering Systems). Two powder-pressed, 3-inch diameter target compositions were employed as sputtering targets, consisting of 70.8 atomic% Nb_2_O_5_+29.2 atomic% Na_2_O (10 atomic% Na), and 84 atomic% Nb_2_O_5_+16 atomic% Na_2_O (5 atomic% Na). The chamber was first pumped down to achieve high vacuum conditions (≈ 2 × 10^− 6^ Torr) via a diffusion pump and a rotary pump. The sample holder was rotated for increased uniformity of the films, and the deposition was carried out in a controlled oxygen and argon atmosphere, with respective gas flow rates of 20:5 (sccm). The sputtering pressure was maintained at 5 mTorr, with an RF-power of 250 W. The deposition rate was about 1.3 nm per minute using the above conditions.

### Optical characterization of sodo-niobate thin films and waveguides

To characterize thin-film loss, we employed prism coupling (Metricon); further details are provided in Supplementary Fig. 3. The measured thin-film propagation loss at a wavelength of 1550 nm was less than 1 dB/cm. Optical dispersion, specifically the refractive index and extinction coefficient, was determined by spectroscopic ellipsometry and validated through model fitting (see Supplementary Fig. 4). The niobate films deposited via RF magnetron sputtering exhibited a refractive index of approximately *n* = 2.1 at 1550 nm.

Additionally, the propagation loss of the fabricated waveguides was characterized using the Fabry–Pérot (FP) interference method (see Supplementary Fig. 5). Following the etching process, the waveguide propagation loss was approximately 4 dB/cm at 1550 nm. Although the waveguides exhibited clear light guiding behaviour after poling (Supplementary Fig. 6), the edge coupler and waveguide dimensions of the poled samples differed from those of the unpoled devices; therefore, the losses are not directly comparable. Further optimization of the waveguide design, along with systematic loss measurements and comprehensive electro-optic characterization, is currently underway and will be reported in a forthcoming publication.

### Thermal poling of sodo-niobate thin films on BF33 glass substrate

We employed a structured thermal poling technique reported by Karam et al.^20^. The sodo-niobate films were sandwiched between an anode (film side) and cathode (substrate side) as shown in Supplementary Fig. 7. The anode was made of a 200 nm thick platinum layer deposited on borosilicate glass and patterned via lithography to form grids with non-conductive cells of 70 × 70 (or 40 × 40) µm^2^ separated by 4 μm wide conductive Pt strips. The cathode was a silicon wafer. The procedure began by heating the poling chamber at a rate of 15 °C per min up to 275 °C. The atmosphere is controlled using a nitrogen gas flow of 6 L per min. Direct current (DC) voltages of 900 V (or 1500 V) were then applied to the sample at a rate of 375 V per min and maintained for 30 min. Lastly, the sample was cooled down to room temperature before switching off the DC supply. An optical microscope image, in Supplementary Fig. 7(c), displays the poled line on the films corresponding to contact regions between the film and conductive Pt strips in. This contrast will be useful for waveguide alignment in the next step.

### Waveguide fabrication procedure

To prepare the waveguide pattern for the etching process, UV contact lithography was employed. Initially, the poled sodo-niobate films on BF33 glass substrate were spin-coated with S1827 positive photoresist. The coated substrate was then heated on a hotplate at 115 °C for 60 s to achieve a resist thickness of 2.7 μm. A light-field mask, featuring straight waveguides with different widths ranging from 1 μm to 20 μm, was aligned onto the substrate. The substrate was subsequently exposed to UV radiation for 13 s at an intensity of 17.2 mW cm^− 2^ using hard-contact mode. After UV exposure, the sample was immersed in an MF-319 developer solution for 1 min and 5 s to develop the resist pattern. The Nb$\:{}_{2}$O$\:{}_{5}$ layer was then etched through argon ion beam milling (Oxford Instruments Plasma Technology Ionfab 300+). Finally, any residual photoresist was removed via O$\:{}_{2}$ plasma etching. The sequential fabrication steps used in the process are illustrated in Supplementary Fig. 8.

## Supplementary Information

Below is the link to the electronic supplementary material.

Supplementary Material 1

## Data Availability

The data for this work are accessible through the University of Southampton Institutional Research Repository [https://eprints.soton.ac.uk/](https:/eprints.soton.ac.uk).
