# Supplementary material for: Sustained poling-induced second-order optical nonlinearity in sodium-doped amorphous niobium oxide waveguides
Source: Sci Rep. 2026 Mar 26;16:15146. doi: 10.1038/s41598-026-45779-5 (PMC13172450; doi:10.1038/s41598-026-45779-5)

Supplementary Information

Sustained Poling-Induced Second-Order Optical Nonlinearity in Sodium-Doped Amorphous Niobium Oxide Waveguides

Sirawit Boonsit[1], Lara Karam[2], Frederic Adamietz[2], Lydie Bourgeois[2], Milos Nedeljkovic[1], Nadege Courjal[3], Marc Dussauze[2], and Ganapathy Senthil Murugan*[1]

[1]Optoelectronics Research Centre, University of Southampton, Southampton, SO17 1BJ, UK

[2]Institut des Sciences Moléculaires, Université de Bordeaux, Talence Cedex 33405, France

[3]FEMTO-ST TEMIS, 15B avenue des Montboucons, Besancon Cedex 25030, France

Supplementary Figure 1: μ **-SHG experimental setup.** Schematic of the experimental setup for μ-SHG measurement of the poled films and waveguides.

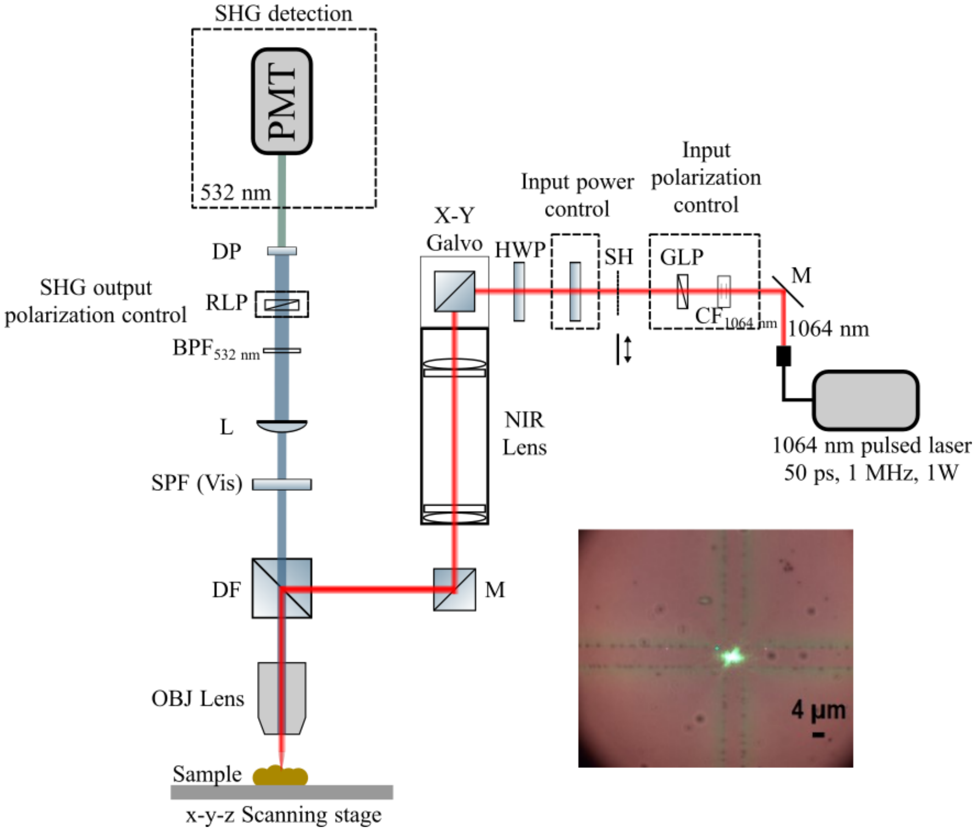

(BPF: Bandpass filter, BS: 50/50 beam splitter, CF: clean-up filter, DF:dichroic filter, DP:depolarizer, GLP:Glan-laser polarizer, HWP:half-wave plate, L:lens, M:mirror, NDF:neutral density filter, OBJ:20x objective lens, PMT: photomultiplier tube, RLP: Rotating linear polarizer, SH:shutter, SPF:short pass filter, X-Y galvo: galvanometer mirrors)

Polarized SHG images were acquired at room temperature using a custom scanning SHG microscope as shown in Supplementary Figure 1. The excitation source was a 1064 nm picosecond laser (Leukos Opera), delivering 50 ps pulses at a 1 MHz repetition rate. The laser linear polarization was controlled using polarizers, specifically Glan-laser polarizer and half-wave plate. Then, the laser beam, with an average power of 450 mW, was focused on the surface of poled thin films using a near-IR 20X objective (Mitutoyo M-PLAN APO, NA 0.4). The back-reflected SHG signal (epi-detection mode) was collected by the same objective lens, and unwanted wavelengths other than 532 nm were filtered out using a short-pass filter and a band-pass filter. Polarization was controlled by a rotating linear polarizer and a depolarizer before the signal reached a photomultiplier tube. Polarized SHG images were recorded with a spatial resolution of 1 μm in both the x and y directions.

Supplementary Figure 2: Extracting μ-SHG data from etched waveguides. a Microscopic images of the chip reveal waveguides at varying distances from the poling electrode. **b** μ-SHG image of poled waveguides at different positions. **c** μ-SHG intensity measured across the poled waveguide. **d** The maximum μ-SHG intensity from the poled waveguide is plotted as a function of distance from the waveguide to the electrode, and compared with that of the poled films (blue solid line).

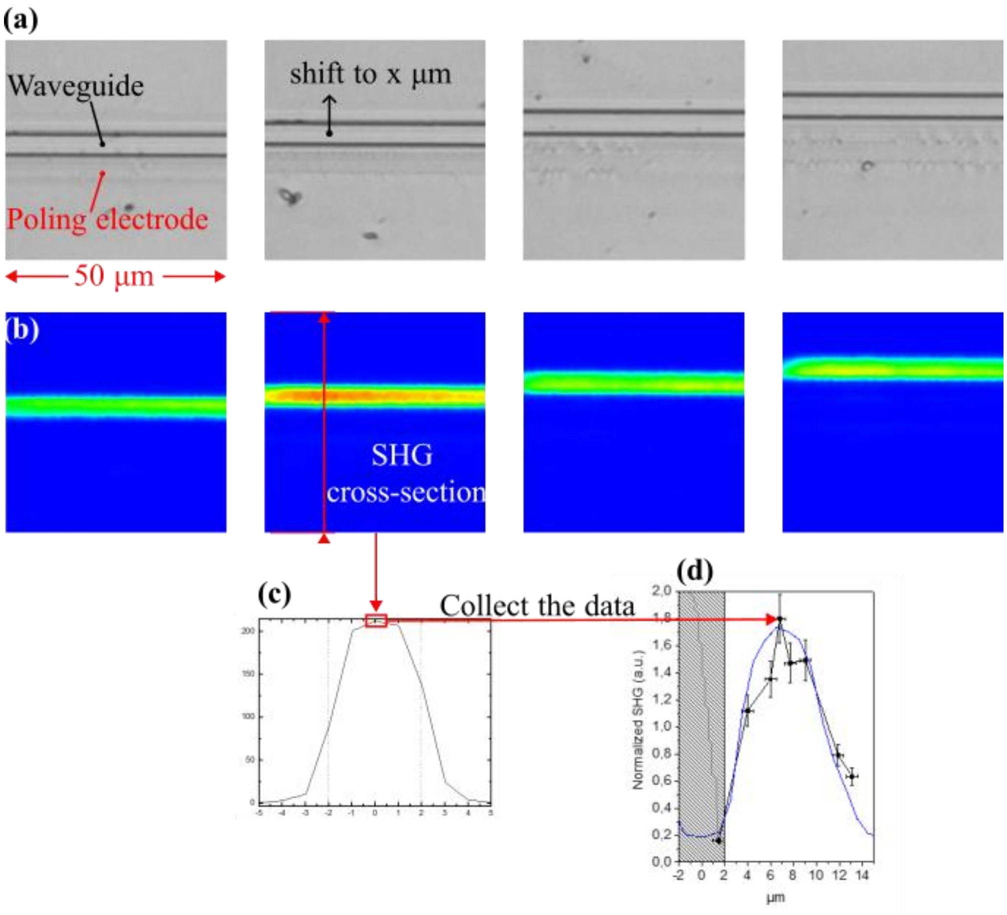

The Supplementary Figure 2 shows our data acquisition method for obtaining μ-SHG of the etched waveguide at various positions from poling electrode. As shown in the optical microscope images (Supplementary Figure 2(a)), the waveguides were aligned at different positions from the poling electrode. The μ-SHG intensity cross-section from the poled waveguide was measured at different positions from the poling electrode, as illustrated in Supplementary Figure 2(b). Supplementary Figure 2(c) shows the μ-SHG intensity measured across the poled waveguide, with its maximum value plotted against the gap distance from the poling electrode in Supplementary Figure 2(d). These values were compared to pre-etching data extracted from Figure 3 in the main text.

Supplementary Figure 3: Optical characterization of sodo-niobate thin films and waveguides. (a) METRICON measurement apparatus for loss measurement (light streak shown at 633 nm wavelength)**. (b)** Measurement of the refractive index and thickness of the film (1550 nm). **(c)** Decay of the guided light streak (black line) with a fitting line (green) at 1550 nm wavelength.

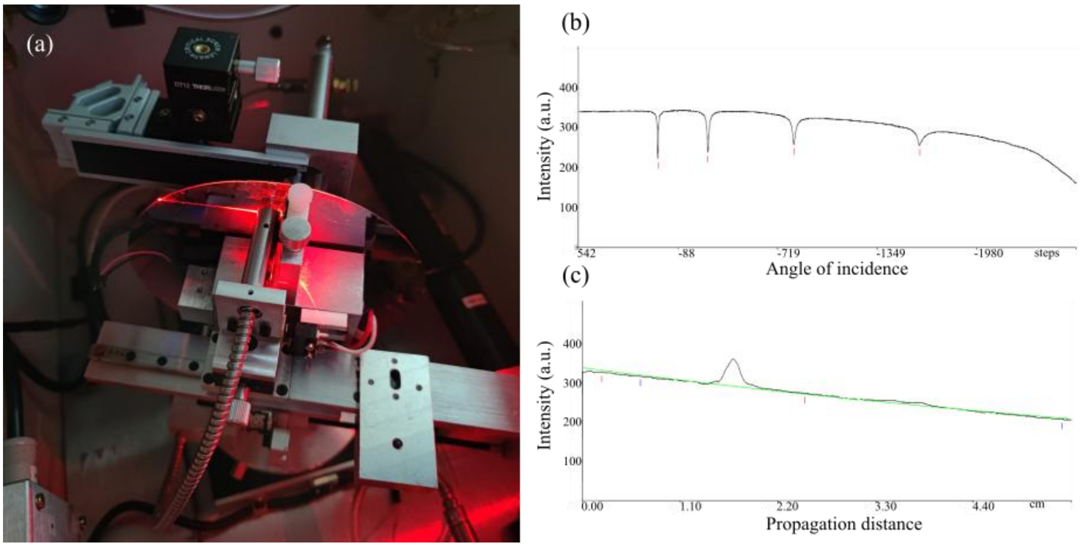

Film propagation losses were measured using prism coupling (METRICON) with a 1550 nm laser, transparent to Nb_2O_5. Guiding was achieved by rotating the coupling angle until a light streak or intensity dip was observed (Supplementary Figure 3b). The propagation loss (dB/cm) was determined by fitting the guided light intensity decay using METRICON software (Supplementary Figure 3c).

Supplementary Figure 4: Ellipsometry data fitting: **(a)** Fit transparent region with Cauchy or Sellmeier model. **(b)** Convert to b-spline and extend fitting to short wavelengths. **(c)** Add a gen-osc for improved accuracy. **(d**) Plot of dispersion (n and κ).

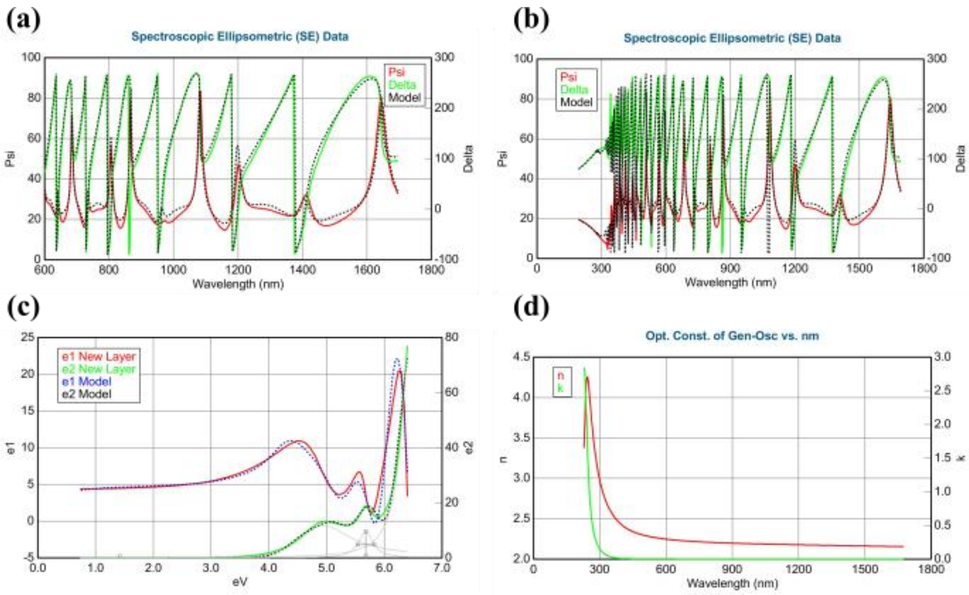

The refractive index, band gap energy, and extinction coefficient of the sodo-niobate thin films were determined using spectroscopic ellipsometry (Woollam M-2000 XI). This technique analyzes changes in light polarization upon reflection and transmission to characterize layer thickness and optical constants. Initial film thickness was determined with a transparent model (Cauchy or Sellmeier), then refined by iteratively fitting a B-spline model to the absorption region in 0.3–0.5 eV steps. Finally, the Kramers-Kronig relation and a Tauc-Lorentz oscillator model were used to refine the optical constants. The niobate films deposited via RF magnetron sputtering exhibited a refractive index of approximately n =2.1 at 1550 nm wavelength.

Supplementary Figure 5: FP loss measurement at 1550 nm: **(a, b)** Optical images of output and input lensed fibers at waveguide facets. **(c)** Raw FP interference data. **(d)** FFT-filtered data showing waveguide cavity resonance with a 60 pm FSR.

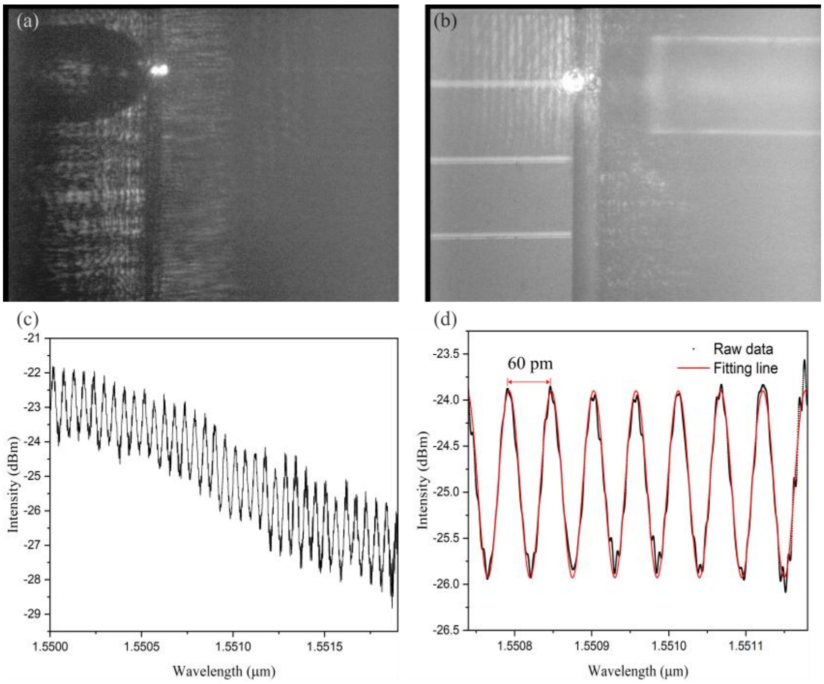

We employed the Fabry-Pérot (FP) method to measure waveguide propagation loss. A continuous wave (CW) laser with an average power of 3 mW was coupled into a single-mode lensed fiber (OZ optics) with a working distance (WD) of $26 \pm 3\ \mu\mathrm{m}$ (NA = 0.09). A top-mounted camera was used to position the fiber tip at the waveguide end facet. The output light was coupled into another lensed fiber (Thorlabs) with a WD of 30 $\mu\mathrm{m}$ (NA=0.2), which leads to the detector module. The wavelength was varied in the range of $1.55\ \mu\mathrm{m} - 1.5519\ \mu\mathrm{m}$ with a step size of 0.1 pm to obtain the interference fringes.

The raw FP spectra typically contain multiple cavity resonances arising from reflections between the waveguide facets and external optical components (e.g., fiber tips). To isolate the primary resonance, we applied a Fast Fourier Transform (FFT) filter to the raw data. The filtered response reveals a single Free Spectral Range (FSR) of approximately 60 pm, consistent with the 1 cm waveguide length. The propagation loss was then extracted by fitting the processed data to Equation.

$$\frac{I_t}{I_0} = \frac{(1-R)^2 e^{-\alpha L}}{(1-Re^{-\alpha L})^2 + 4e^{-\alpha L} R sin^2(2\pi n_g L/\lambda)}$$

where, I_t and I_0 represent the transmitted and incident light intensities, respectively. L denotes the waveguide length, and α represents the waveguide propagation loss.

Supplementary Figure 6: Waveguiding in (a) unpoled and (b) poled waveguides

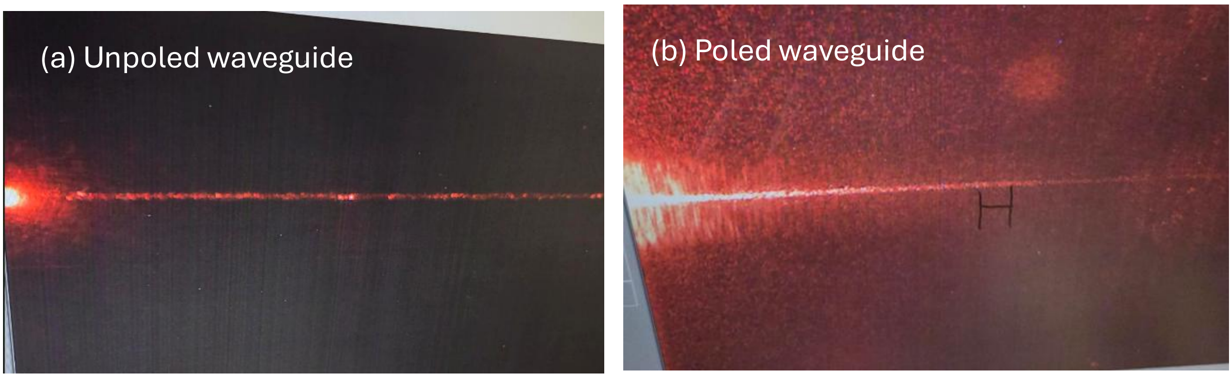

The images above clearly demonstrate waveguiding at 633 nm in both the unpoled and poled samples. The faster decay of the streak in the poled waveguide provides evidence of higher loss, which requires further investigation and optimization. The high loss could also be attributed to the electronic absorption edge and associated Urbach tails shifting to longer wavelengths due to poling-related compositional changes. Therefore, measurements at longer wavelengths may yield better results and will be carried out in due course.

Supplementary Figure 7: Experimental setup for thermal poling on sodo-niobate thin film. (a) Schematic cross-section of the thermal poling setup used to imprint patterns onto a sodo-niobate thin film deposited on a BF33 glass substrate. Optical microscope image of (**b)** the poling electrode, highlighting the conductive zones (with electrode contact) and non-conductive zones (without electrode contact), (**c)** the poled sodo-niobate film showing electrode pattern due to refractive index contrast after the thermal poling. (**d)** Diagram of the thermal poling with charge migration mechanism in the thin film.

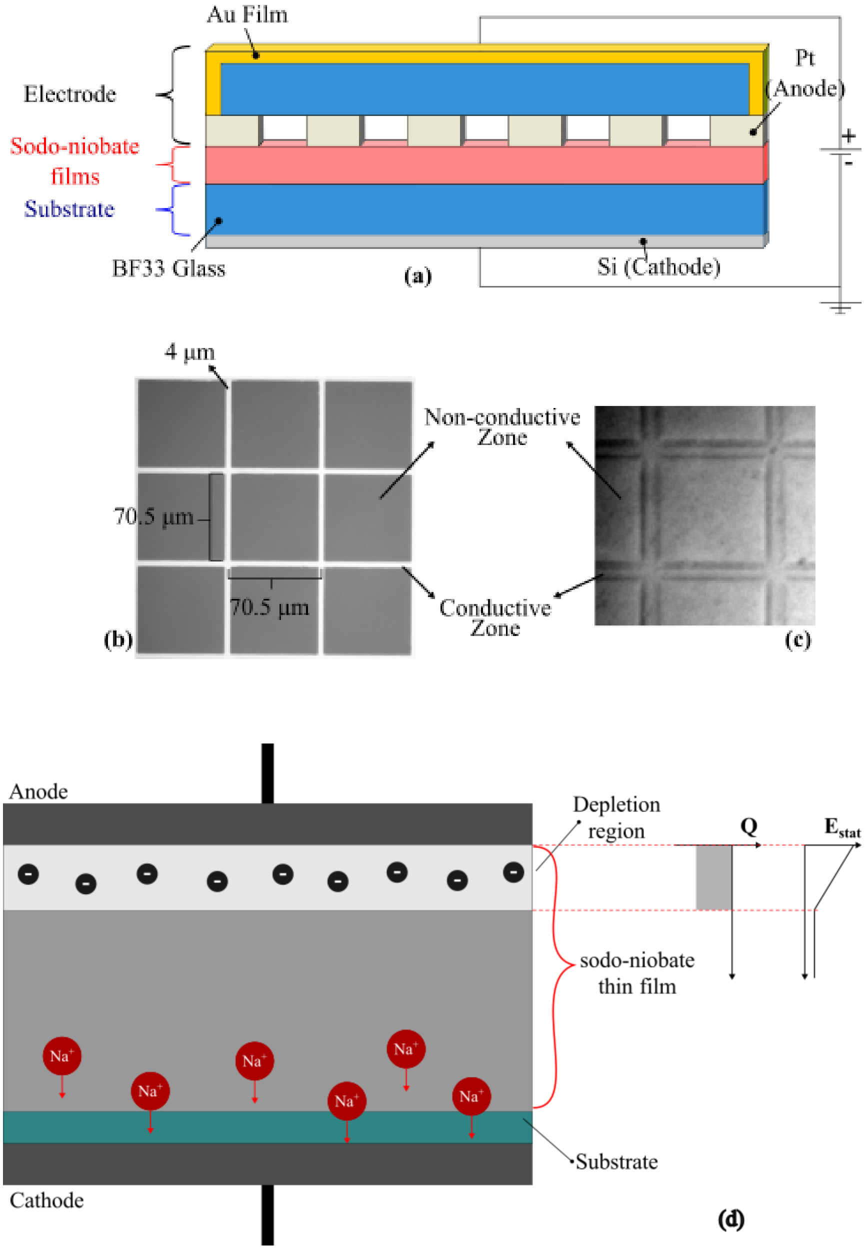

The sodo-niobate thin film was placed between a platinum anode and a silicon wafer cathode, with a borosilicate glass slide supporting the film. The chamber was evacuated to low pressure before introducing nitrogen gas. Humidity was carefully monitored to prevent moisture during the poling process. The chamber was then heated to approximately 275 °C to increase the kinetic energy of the positive charges. High

voltage of 900 V or 1500 V were applied to the sample, driving sodium cations to migrate from the anode to the cathode, resulting in a depletion layer as illustrated in Supplementary Figure 6(d). The chamber containing the sample was then allowed to cool to room temperature before the electric field was removed. This process generates a static electric field (E_{stat}) within the depletion layer, which contributes to the induction of SONL properties in the film, following this equation: $\chi^{(2)} = 3. \chi^{(3)} E_{stat}$.

Supplementary Figure 8: Schematic illustration of the waveguide fabrication process on thermally poled sodo-niobate thin films. (a) Deposition of $Na_2O:Nb_2O_5$ thin film followed by thermal poling resulting the refractive index contrast on the films. (**b)** Photoresist patterns aligned to poled regions (with electrodes contact) by UV lithography. (**c)** Dry etching is performed via argon ion beam milling. (**d) and (e)** Fully etched and partially etched poled sodo-niobate waveguides after resist removal.

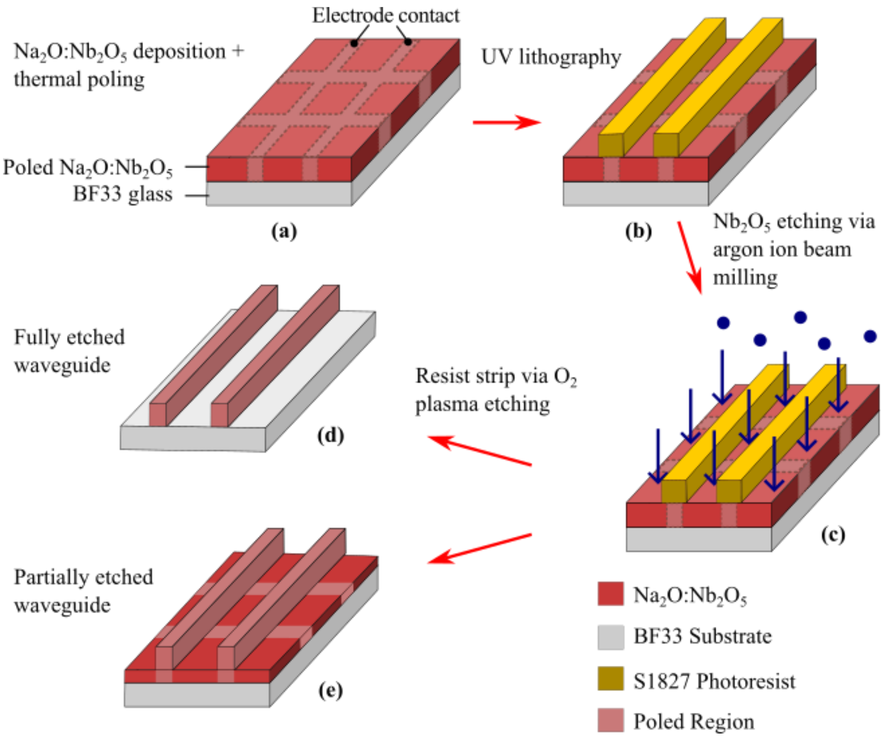

Supplement: Supplementary file 1 — Supplementary Material 1 [file 41598_2026_45779_MOESM1_ESM.pdf]
